# Successful Preservation of a Developing Unerupted Tooth Associated With Dentigerous Cyst in a Child: A Case Report

**DOI:** 10.1155/crid/6972721

**Published:** 2025-09-05

**Authors:** Janaki Shaji, Parvathy Kumaran, Ravi Veeraraghavan, R. Balagopal Varma, Mahija Janardhanan, J. Suresh Kumar, Arun Mamachan Xavier, Malini Venugopal, T. Nishna

**Affiliations:** ^1^Department of Pediatric and Preventive Dentistry, Amrita School of Dentistry, Amrita Vishwa Vidyapeetham, AIMS, Kochi, Kerala, India; ^2^Department of Oral and Maxillofacial Surgery, Amrita School of Dentistry, Amrita Vishwa Vidyapeetham, AIMS, Kochi, Kerala, India; ^3^Department of Oral & Maxillofacial Pathology, Amrita School of Dentistry, Amrita Vishwa Vidyapeetham, AIMS, Kochi, Kerala, India

**Keywords:** dentigerous cyst, marsupialization, obturator, tooth eruption

## Abstract

Dentigerous cysts (DCs) are odontogenic cysts typically associated with the crowns of unerupted or impacted teeth and are usually discovered when they reach large sizes or get infected. Although common in adults, their occurrence in children are rare. Most cases in children are incidentally discovered on routine radiographic examination of infected deciduous molars. Treatment methods vary based on the size and extend of the lesion, with enucleation and marsupialization being the commonly employed approaches. Despite the variation in techniques, the overall prognosis and treatment outcomes are generally favorable. This case report describes the successful conservative management of a mandibular DC in an 8-year-old female through marsupialization in conjunction with a custom-made acrylic obturator.

## 1. Introduction

Dentigerous cysts (DCs) are odontogenic cysts of developmental origin, most commonly associated with crowns of unerupted teeth, that expands the dental follicle and is typically attached to the cementoenamel junction [1]. They occur predominantly in the mandible, accounting for over 70% of cases. Over twice as prevalent in males than females, they seldom occur in children [2].

DCs are typically asymptomatic and often discovered incidentally as well-defined radiolucency surrounding the crown of an unerupted tooth, most often the mandibular third molar, on panoramic radiographs taken as part of routine dental procedures or while investigating the reason behind delayed tooth eruption. Histologically, they are lined by nonkeratinized stratified squamous epithelium resembling reduced enamel epithelium [3].

Management of DCs depends on cyst size, location, and patient age. The standard treatment for DCs typically involves enucleation along with the extraction of the affected tooth [4]. However, in pediatric patients, conservative approaches such as marsupialization or decompression are often preferred to preserve the developing tooth bud. Marsupialization, first described by Partsch in 1892, involves resecting the cyst's outer wall, exposing it, and allowing the remaining capsule to develop into oral mucosa. Introduced by Thoma in 1958, decompression is a variation that involves surgically draining the cyst with a catheter or drain that is sutured to the mucosa [5].

This case study illustrates the conservative management of a DC in an 8-year-old patient by marsupialization combined with a custom acrylic obturator, which successfully preserves adjacent structures and promotes tooth eruption.

## 2. Case Presentation

An 8-year-old female presented with a complaint of swelling in the right lower jaw, noticed by her parents 2 weeks ago. The swelling was described as gradually increasing in size, asymptomatic, with no history of pain, discharge, or trauma. Medical history was noncontributory.

**Figure 1:**
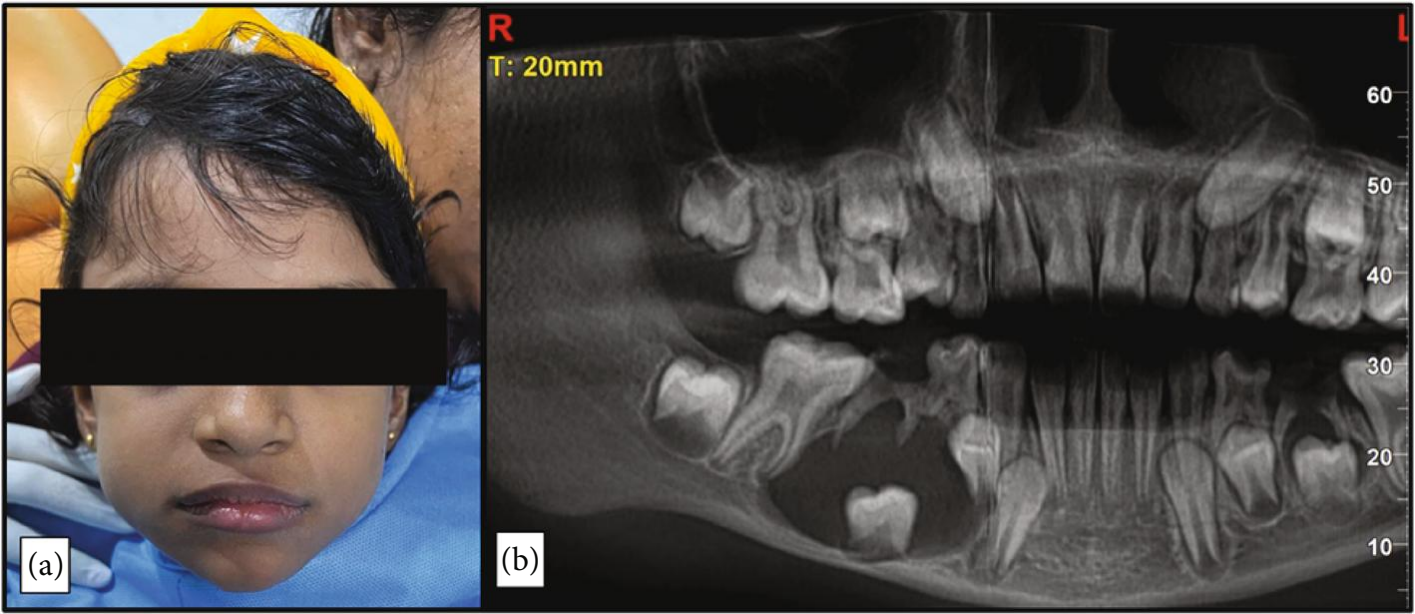
(a) Extraoral appearance showing swelling on the right lower jaw. (b) OPG showing radiolucent lesion in relation to unerupted second premolar.

On extraoral examination, a diffused oval swelling measuring approximately 2 cm in diameter was noticed on the right side of the face extending from the body of the mandible to almost the ramus with limited mouth opening. Palpation revealed a nontender, nonpulsatile, bony hard swelling with no local rise in temperature (Figure 1a). The overlying skin appeared normal. On intraoral examination, a firm, nontender swelling was noted in the right buccal vestibule extending till the retromolar pad causing vestibular obliteration. The overlying mucosa was intact, with no signs of ulceration or discharge. Grossly decayed lower right deciduous second molar was noted. No vitality test was carried out.

The orthopantomogram (OPG) revealed a unilocular, radiolucent lesion with a well-defined radiopaque border associated with unerupted mandibular right second premolar (Figure 1b).

**Figure 2:**
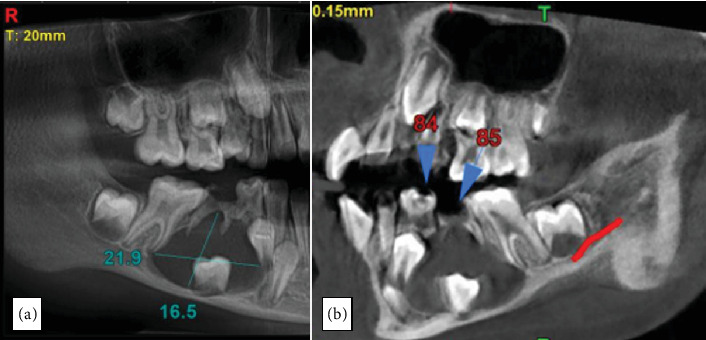
CBCT showing (a) extent of the lesion and (b) its relation with mandibular canal.

CBCT showed a well-defined hypodensity around the crown of the lower right second premolar, measuring 21.9 × 16.9 mm (Figure 2). Buccal cortical thinning and gross expansion of the bone were evident.

Additionally, the mandibular canal was pushed downwards, crossing the unerupted tooth closely through the center of the lesion with its superior margin being interrupted posteriorly. Fine needle aspiration yielded a clear yellow fluid.

Based on the clinical and radiographic findings, the differential diagnosis considered were a radicular cyst arising from periapical region of mandibular right second deciduous molar and a DC associated with unerupted mandibular right second premolar. However, due to its large size and association with crown of unerupted tooth, the lesion was provisionally diagnosed as DC.

Taking into account the child's age, the size of lesion, and desire to preserve the developing permanent tooth, a conservative treatment plan using marsupialization was considered. The procedure aimed to decompress the cystic cavity and guide the natural eruption of the unerupted tooth.

Following the administration of local anesthesia, the grossly decayed lower right deciduous second molar was extracted. The cystic cavity was accessed through the extraction socket, and marsupialization was performed by creating a surgical window at the socket site, allowing decompression of the cyst. Curettage of the cystic cavity was carried out carefully to remove debris while ensuring preservation of the underlying developing permanent tooth bud. The socket was thoroughly irrigated with normal saline and 0.2% povidone–iodine solution to minimize the microbial load. A portion of the cystic lining was excised and submitted for histopathological examination.

**Figure 3:**
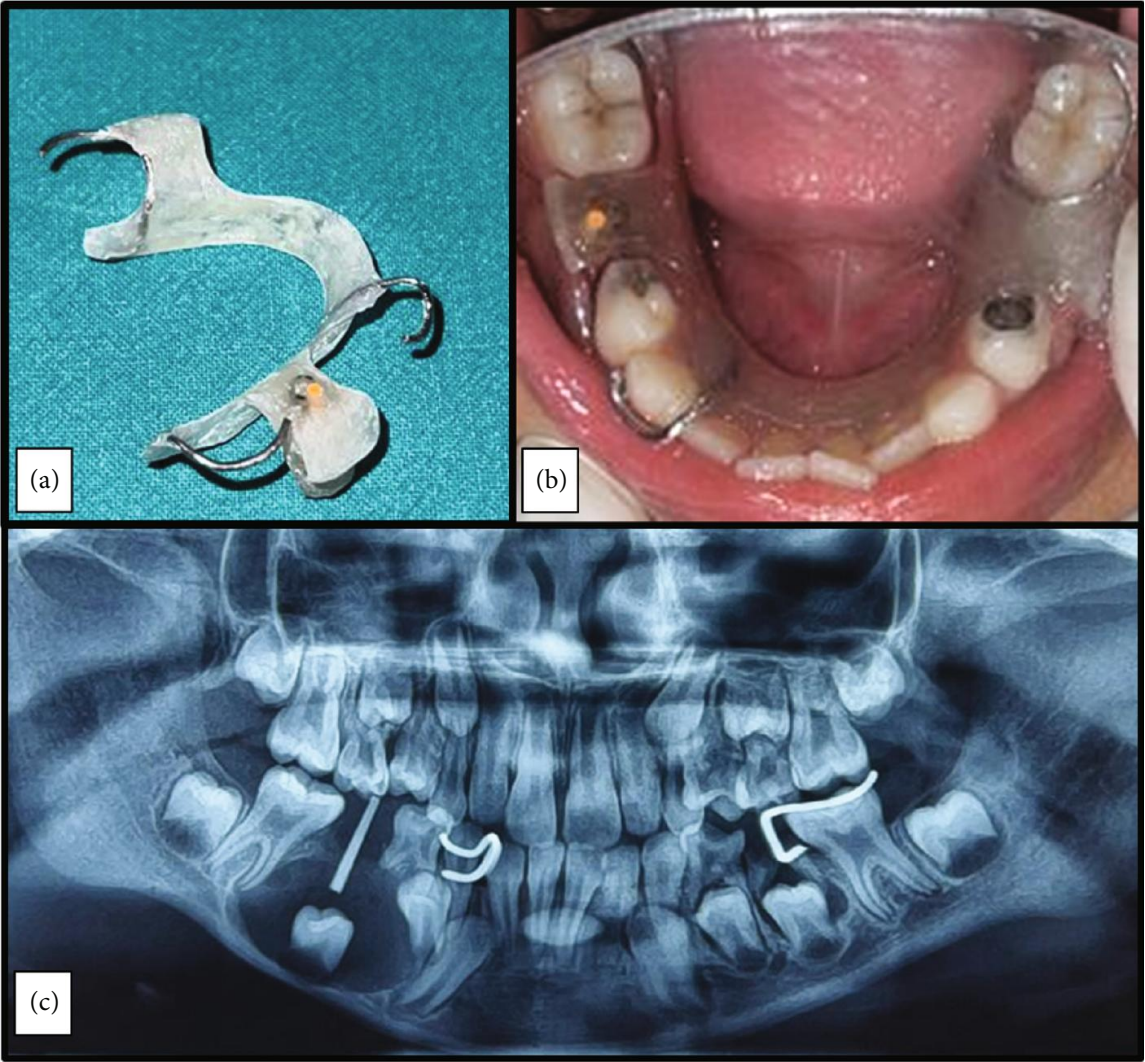
(a) Custom-made acrylic resin obturator, (b) appliance delivery and (c) postoperative OPG.

An impression was made for the fabrication of custom acrylic resin obturator (Figure 3a,b), which was delivered on the same day of the procedure to preserve the surgical site during the healing process; facilitate ongoing decompression, for space maintenance; and to guide the successor tooth. It consisted of C clasps on both sides, acrylic plate and extension in to the extraction socket. A radiopaque gutta percha embedded into the acrylic extension of the obturator helped to evaluate the level of the unerupted tooth postoperatively (Figure 3c).

The patient was instructed to remove the appliance twice daily during tooth brushing to irrigate the cystic cavity with mouthwash and to subsequently reposition the appliance intraorally to maintain decompression and prevent premature closure of the marsupialization window for a period of 2 months. The patient was also scheduled for periodic clinical and radiographic follow-up to monitor healing, reduction in cyst size, and eruption of the permanent tooth.

**Figure 4:**
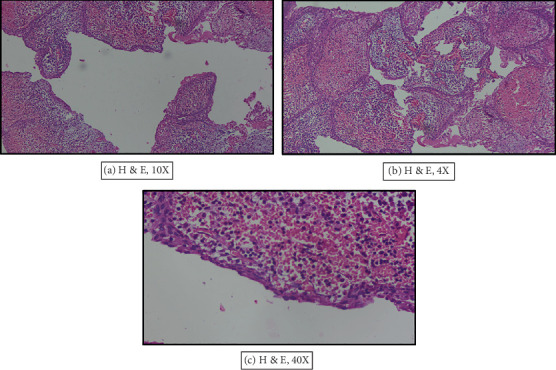
Histopathological findings of the lesion: (a) cystic lumen showing lumen, epithelial lining, and connective tissue capsule; (b) hyperplasia as interconnecting epithelial strands with connective tissue stroma entrapped within giving an arcading pattern; and (c) thin epithelium resembling reduced enamel epithelium.

Histopathologically, tissue sections from the specimen showed a cystic lesion, with lumen, epithelial lining, and connective tissue capsule. The epithelial lining was composed of nonkeratinized stratified squamous epithelium and shows hyperplasia as interconnecting epithelial strands with connective tissue stroma entrapped within giving an arcading pattern. The connective tissue showed a dense infiltrate of chronic inflammatory cells, predominantly lymphocytes. At areas where the inflammatory infiltrate was minimal, the epithelium was thin, composed of two to four layers of flattened cells resembling reduced enamel epithelium, which confirmed the diagnosis of a DC (Figure 4).

The patient was recalled every 2 weeks for clinical evaluation and trimming of the obturator to accommodate healing tissue. The surgical site appeared clean on the follow-up visit. Oral hygiene instructions were reinforced at every visit.

**Figure 5:**
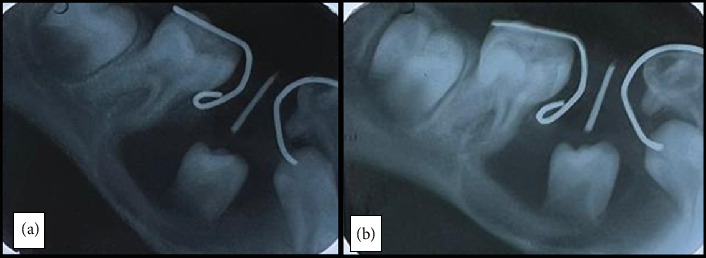
Postoperative radiographs at (a) 4 and (b) 8 weeks, respectively.

Intraoral periapical radiographs taken during the follow-up visits (Figure 5) showed a significant reduction in the size of the radiolucency, suggesting successful decompression after marsupialization. There was visible bone regeneration, especially in the area surrounding the old cystic cavity, indicating that the bone was actively mending and remodeling.

The obturator stayed in position, successfully preserving the area and bolstering the permanent premolar eruption channel.

**Figure 6:**
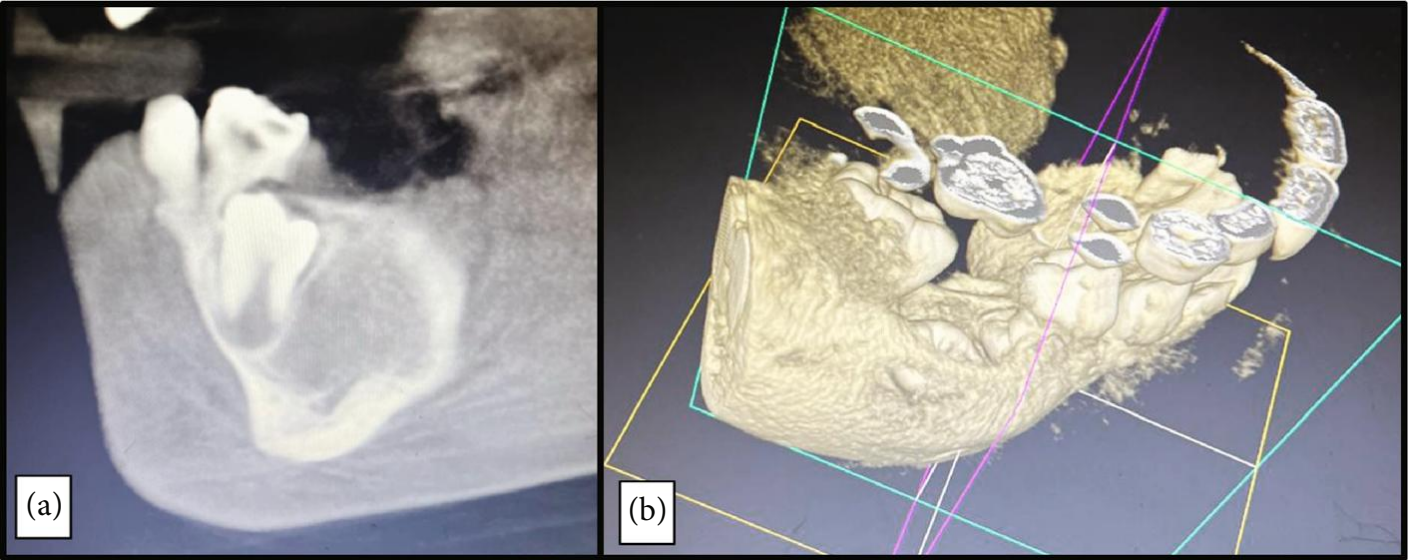
(a) Postoperative CBCT after 3 months and (b) 3D reconstructed image during the same period.

A subsequent CBCT scan at 3-month follow-up showed that the radiolucency had significantly decreased (Figure 6). Early indications of tooth erupting into the oral cavity were observed, along with new bone formation.

**Figure 7:**
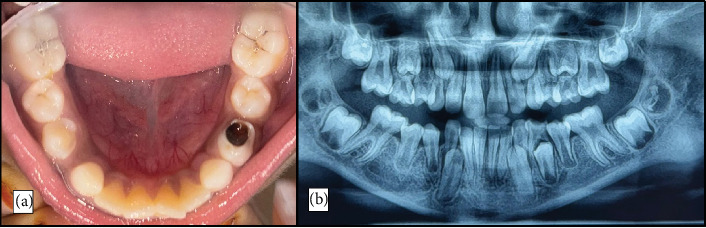
(a) Postoperative intraoral image and (b) OPG after 12 months.

OPG taken at the 12-month follow-up showcased complete resolution of previously noted radiolucent lesion and the successful eruption of the mandibular right second premolar (Figure 7).

This case exemplifies the importance of a conservative approach in managing odontogenic cysts. Marsupialization combined with a custom acrylic obturator offers excellent outcomes in pediatric patients, minimizing surgical morbidity while preserving the developing dentition. Early diagnosis, appropriate imaging, and meticulous and tailored treatment planning were key factors that helped achieve a successful outcome in this case.

## 3. Discussion

DCs are the second most prevalent type of odontogenic cysts, following cysts of periapical origin. They originate from reduced enamel epithelium during the development of the crown and are more often seen in the mandible as opposed to the maxilla and show a prevalence of approximately 20%, with marginally elevated occurrence in males, typically during the early years of adulthood [6]. They are typically solitary, but multiple cysts can occur in association with syndromes like Gardner's syndrome, mucopolysaccharidosis, Maroteaux–Lamy syndrome, and basal cell nevus syndrome.

DCs are often asymptomatic and may go unnoticed for years. Several mechanisms have been proposed for their histogenesis, including pressure exerted by an erupting tooth on the follicle, leading to venous obstruction and transudation of serum, resulting in cyst formation. Alternatively, inflammation from a nonvital deciduous predecessor may also contribute to cyst development around the permanent successor. Radiographically, they are revealed as a well-defined, unilocular, radiolucency enveloping crown of an unerupted tooth [7] and are present in three distinct patterns, namely, central variety, where the cyst surrounds the crown symmetrically; lateral variety, where the cyst enlarges along one side of the crown; and the circumferential variety, in which the cyst envelops the crown and extends along the root surface [8]. In larger lesions, clinical signs like facial asymmetry may be present, as these cysts can expand significantly.

In this case, diagnosis was challenging as the cystic lesion was mimicking a radicular cyst arising from the periapical region of the mandibular right deciduous second molar. A final diagnosis of DC associated with unerupted mandibular right second premolar was determined based on histopathological findings.

The choice of surgical method for management of DC is influenced by numerous factors, including the patient's age, size of the cyst, its extent of invasion, and functional and aesthetic significance of the affected tooth. If left untreated, it has the potential to develop into a painful, aggressive lesion that results in complications like alveolar bone expansion, tooth displacement, severe root resorption, and pathologic bone fracture. Long-standing cases may also lead to the development of ameloblastomas and malignancies like mucoepidermoid carcinoma. [9].

Marsupialization of large DCs is a successful therapy when compared to enucleation as it decreases the damage without endangering neighboring nerves or developing tooth germs. A systematic review and meta-analysis conducted in 2021 assessed four retrospective cohort studies involving DCs associated with lower premolars. It revealed that patients younger than 10 years had a higher odds ratio for tooth eruption postsurgery, whereas those older than 10 years were less likely to experience such outcomes [10].

While both marsupialization and decompression are considered conservative and effective in the management of cysts, decompression has certain drawbacks. These include the inability to examine the entire lesion histologically due to retained pathological tissue, prolonged treatment duration, the need for consistent patient and parental compliance, and the requirement for regular clinical monitoring. Occasionally, a second surgical intervention might be necessary if the decompression device dislodges prematurely, the cyst does not resolve completely, or the histopathology fails to confirm the suspected diagnosis [11].

Conservative management of DCs in pediatric patients, particularly through marsupialization combined with a custom acrylic obturator, proves to be a highly effective and minimally invasive approach. In this case, the patient and caregivers were initially anxious about the swelling and treatment but felt reassured after understanding the conservative approach. They were satisfied with the outcome and experienced minimal discomfort during recovery. It facilitated spontaneous tooth eruption and bone regeneration while avoiding extensive surgical intervention, thereby preserving the developing permanent tooth. Early diagnosis and individualized treatment planning are essential for achieving successful outcomes in managing such cystic lesions in children.

## Data Availability

The data that support the findings of this case are available on request from the corresponding author. The data are not publicly available due to privacy or ethical restrictions.
