# Supplementary material for: Successful Preservation of a Developing Unerupted Tooth Associated With Dentigerous Cyst in a Child: A Case Report
Source: Case Rep Dent. 2025 Sep 5;2025:6972721. doi: 10.1155/crid/6972721 (PMC12431804; doi:10.1155/crid/6972721)
Supplement: Supporting Information — Additional supporting information can be found online in the Supporting Information section. CARE checklist has been included as a supporting information. [file 6972721.f1.docx]

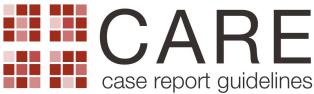
**CARE Checklist (2013) of information to include when writing a case report
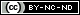
**

**Topic Item Checklist item description Reported on Page**

**Title 1** The words “case report” should be in the title along with the area of focus 1

**Key Words 2** 2 to 5 key words that identify areas covered in this case report 1

**Abstract 3a** Introduction—What is unique about this case? What does it add to the medical literature? 1

**3b** The main symptoms of the patient and the important clinical findings 1

**3c** The main diagnoses, therapeutics interventions, and outcomes 1

**3d** Conclusion—What are the main “take-away” lessons from this case? 1

**Introduction 4** One or two paragraphs summarizing why this case is unique with references 7

**Patient Information 5a** Demographic information and other patient specific information 8

**5b** Main concerns and symptoms of the patient 8

**5c** Medical, family, and psychosocial history including relevant genetic information (also see timeline). 8

**5d** Relevant past interventions and their outcomes 8,9,10

**Clinical Findings 6** Describe the relevant physical examination (PE) and other significant clinical findings 8,9,10

**Timeline 7** Important information from the patient’s history organized as a timeline 8

# Diagnostic Assessment

**Therapeutic Intervention**

**Follow-up and Outcomes**

**8a** Diagnostic methods (such as PE, laboratory testing, imaging, surveys) 9,10,11,12

**8b** Diagnostic challenges (such as access, financial, or cultural) 8,9,16

**8c** Diagnostic reasoning including other diagnoses considered 8,9,16

**8d** Prognostic characteristics (such as staging in oncology) where applicable 8

**9a** Types of intervention (such as pharmacologic, surgical, preventive, self-care) 10,11,13,14

**9b** Administration of intervention (such as dosage, strength, duration) 10,11,13,14

**9c** Changes in intervention (with rationale) 10,11

**10a** Clinician and patient-assessed outcomes (when appropriate) 13,14,15

**10b** Important follow-up diagnostic and other test results 13,14

**10c** Intervention adherence and tolerability (How was this assessed?) 13,14

**10d** Adverse and unanticipated events 16

**Discussion 11a** Discussion of the strengths and limitations in your approach to this case 15,16,17

**11b** Discussion of the relevant medical literature 15,16

**11c** The rationale for conclusions (including assessment of possible causes) 15,16,17

**11d** The primary “take-away” lessons of this case report 17

**Patient Perspective 12** When appropriate the patient should share their perspective on the treatments they received 17

**Informed Consent 13** Did the patient give informed consent? Please provide if requested . . . . . . . . . . . . . . . . . . . . . . . . . . . . . . . . . . . . . .**Yes**
